
JOURNAL INFORMATION
==============================
NLM Title Abbreviation: Wirtschaftsdienst
Journal ID: Wirtschaftsdienst
NLM Journal ID: 101086612

Wirtschaftsdienst (Hamburg, Germany : 1949)

ISSN: 0043-6275

ARTICLE INFORMATION
==============================
PMCID: PMC7813606
PMID: 33487768
DOI: 10.1007/s10273-021-2818-4
Article ID: 2818
Article version: 1
Subjects: Zeitgespräch

Arbeitsmarkteffekte der Corona-Krise — Sind Berufsgruppen mit niedrigen Einkommen besonders betroffen?

Buch Tanja Tanja.Buch@iab.de

Hamann Silke Silke.Hamann2@iab.de

Niebuhr Annekatrin Annekatrin.Niebuhr@iab.de

Roth Duncan Duncan.Roth@iab.de

Sieglen Georg Georg.Sieglen@iab.de

grid.494029.2 0000 0001 2237 4175 Regionales Forschungsnetz, Inst. f. Arbeitsmarkt- und Berufsforschung der BA, Regensburger Straße 100, 90478 Nürnberg, Deutschland
Electronic publication date: 2021 Jan 19
Print publication date: 2021
Volume: 101
Issue: 1
First page: 14
Last page: 17
Copyright: © Der/die Autor(en) 2021
License: Open Access: Dieser Artikel wird unter der Creative Commons Namensnennung 4.0 International Lizenz (https://creativecommons.org/licenses/by/4.0/deed.de) veröffentlicht.
Open Access wird durch die ZBW — Leibniz-Informationszentrum Wirtschaft gefördert.
License URL: https://creativecommons.org/licenses/by/4.0/

issue-copyright-statement: © The Author(s) 2021

==============================
Dr. Tanja Buch, Silke Hamann, Prof. Dr. Annekatrin Niebuhr, Dr. Duncan Roth und Georg Sieglen sind wissenschaftliche Mitarbeiter:innen am Institut für Arbeitsmarkt- und Berufsforschung.

Im Frühjahr des Jahres 2020 ist es infolge der Corona-Krise zu einem erheblichen Anstieg der Arbeitslosigkeit gekommen. Insbesondere in Wirtschaftsbereichen, die zu einer Einschränkung ihrer Geschäftstätigkeit gezwungen waren, wie dem Gastgewerbe oder der Unterhaltungsindustrie, kam es zu einem deutlichen Anstieg der Arbeitslosigkeit (Gehrke und Weber, 2020). Mit der spezifischen Betroffenheit einzelner Branchen geht einher, dass bestimmte Berufe stärker von der Krise betroffen sind als andere. Zudem sind z. B. im Gastgewerbe überwiegend Beschäftigte tätig, die bereits vor der Krise eher geringe Löhne erzielt haben. Die Corona-Krise droht somit, bereits bestehende soziale Ungleichheiten zu verschärfen (Hövermann und Kohlrausch, 2020). In diesem Beitrag zeigen wir zunächst, in welchen Berufen die Zugänge in Arbeitslosigkeit coronabedingt besonders gestiegen sind, und untersuchen anschließend, ob es einen Zusammenhang zwischen der Betroffenheit eines Berufs und der Höhe des beruflichen Entgelts gibt.

## Unterschiedliche Betroffenheit von Berufsgruppen

Für die Untersuchung unterscheiden wir 140 Berufsgruppen gemäß der Klassifikation der Berufe 2010. Um die Betroffenheit der Berufsgruppen durch die Corona-Pandemie abzubilden, verwenden wir Zugangsraten in Arbeitslosigkeit. Zur Berechnung der Zugangsraten werden zunächst die Übergänge aus abhängiger Beschäftigung am ersten Arbeitsmarkt in Arbeitslosigkeit im Zeitraum April bis November 2020 ermittelt. Die Summe der Übergänge wird auf die Zahl der sozialversicherungspflichtig Beschäftigten in der jeweiligen Berufsgruppe im Juni 2019 bezogen, um Größenunterschiede zwischen den Gruppen zu kontrollieren. Entsprechend berechnen wir die Zugangsrate für 2019 und bilden die coronabedingte Veränderung der Zugänge in Arbeitslosigkeit durch die Differenz der beiden Zugangsraten ab. Diesem Corona-Effekt liegt die Annahme zugrunde, dass sich Übergänge in Arbeitslosigkeit ohne die Krise so dargestellt hätten wie im Jahr 2019. Zu beachten ist, dass diese Größe lediglich die Veränderungen auf der Zugangsseite abbildet. Die Abgänge aus Arbeitslosigkeit in Beschäftigung lassen sich differenziert nach Berufen gegenwärtig noch nicht bestimmen.

Für einige Berufe sind auf der Grundlage der Zugangsraten keine signifikanten Auswirkungen des coronabedingten Arbeitsmarktschocks festzustellen, weil sie in Bereichen der Wirtschaft angesiedelt sind, auf die sich die Krise kaum ausgewirkt hat. Das gilt z. B. für die Verwaltungsberufe und für Tätigkeiten im Bereich der Versicherungs- und Finanzdienstleistungen. Für einzelne Berufsgruppen verlief die Entwicklung sogar etwas günstiger als im Vorjahreszeitraum, so unter anderem für Mathematiker:innen, Statistiker:innen und für Berufe im Umweltmanagement und der Umweltberatung.

Im Gegensatz dazu sind einige Berufsgruppen weit überdurchschnittlich von den Auswirkungen der Krise betroffen (vgl. Abbildung 1). Über alle Berufe hinweg ist die Zugangsrate in Arbeitslosigkeit gegenüber 2019 um 3,5 Personen je 1.000 Beschäftigte gestiegen. Das entspricht fast 117.000

**Dr. Tanja Buch**, **Silke Hamann**, **Prof. Dr. Annekatrin Niebuhr**, **Dr. Duncan Roth** und **Georg Sieglen** sind wissenschaftliche Mitarbeiter:innen am Institut für Arbeitsmarkt- und Berufsforschung.

Abbildung 1

**Krisenbedingter Anstieg der Übergänge von Beschäftigung in Arbeitslosigkeit**

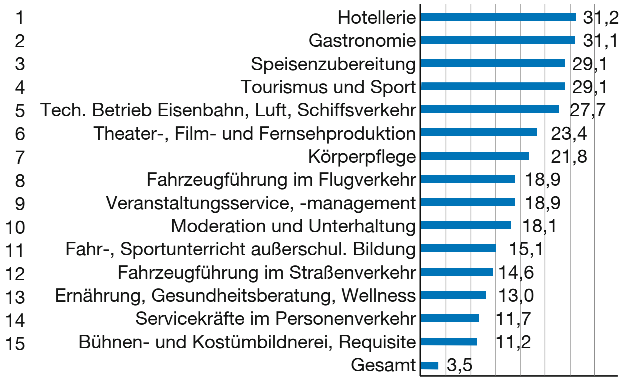

Anmerkungen: Dargestellt ist für die 15 am stärksten betroffenen Berufsgruppen die Differenz zwischen der Summe der Übergänge aus abhängiger Beschäftigung in Arbeitslosigkeit in den Monaten April bis November 2020 und dem entsprechenden Wert aus dem Jahr 2019 pro 1.000 Beschäftigte.

Quelle: Statistik der Bundesagentur für Arbeit; eigene Berechnungen.

Abbildung 2

**Zusammenhang zwischen dem Corona-Effekt und dem Medianentgelt**

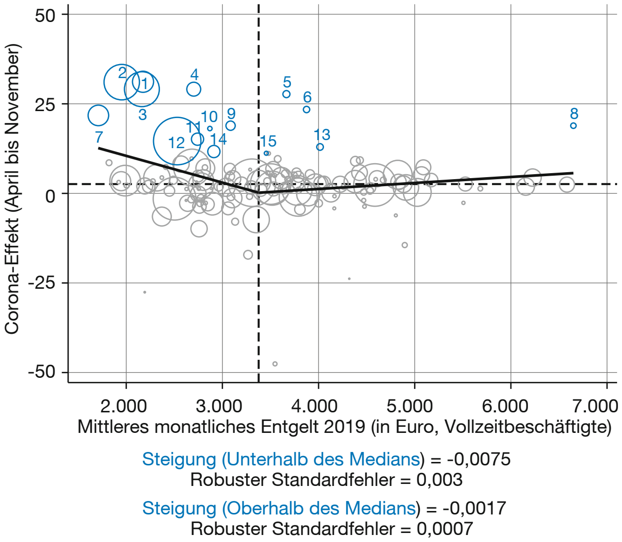

Anmerkungen: Dargestellt sind die 140 nicht-militärischen Berufsgruppen der Klassifikation der Berufe aus dem Jahr 2010. Die Größe der Kreise zeigt die Größe der jeweiligen Berufsgruppe gemessen an der Zahl der Beschäftigten im Juni 2019. Die nummerierten blauen Kreise sind die in Abbildung 1 aufgeführten Berufsgruppen. Die gestrichelten Linien geben den Median der jeweiligen Variablen an. Die durchgezogenen Linien stellen den geschätzten Zusammenhang zwischen dem Corona-Effekt und dem Medianentgelt für Berufsgruppen unterhalb bzw. oberhalb des mittleren Medianentgelts dar. Die Schätzung ist mit der Zahl der Beschäftigten in den jeweiligen Berufsgruppen gewichtet. Es werden robuste Standardfehler berechnet.

Quelle: Statistik der Bundesagentur für Arbeit; eigene Berechnungen.

zusätzlichen Arbeitsplatzverlusten im Zuge der Corona-Krise zwischen April und November 2020. Bei den Gastronomie- und Hotellerieberufen fiel der Kriseneffekt dagegen etwa um den Faktor 9 stärker aus. In beiden Berufsgruppen zusammen sind damit coronabedingt mehr als 22.000 zusätzliche Übergänge in Arbeitslosigkeit zu verzeichnen. Stark betroffen sind daneben vor allem Berufsgruppen, die unterschiedlichen Bereichen der Freizeitgestaltung zuzuordnen sind, sowie verschiedene Verkehrsberufe.

## Sind geringer entlohnte Berufe stärker von Übergängen in Arbeitslosigkeit betroffen?

Im Folgenden gehen wir der Frage nach, inwiefern sich das Ausmaß coronabedingter Übergänge in Arbeitslosigkeit zwischen niedriger und höher entlohnten Berufen unterscheidet. Dafür stellen wir in Abbildung 2 für alle Berufsgruppen die Höhe des Corona-Effekts dem monatlichen Medianentgelt gegenüber. Hierbei zeigt sich, dass ein Großteil der in Abbildung 1 dargestellten Berufsgruppen mit hoher Betroffenheit (blaue Kreise mit entsprechender Nummerierung) am unteren Ende der Medianlohnverteilung liegt. Dazu gehören insbesondere die vier Berufsgruppen mit den höchsten Corona-Effekten – Berufe in der Hotellerie, Gastronomie, Speisezubereitung sowie im Bereich Tourismus und Sport. Ein ausgeprägter Corona-Effekt, der mit einer vergleichsweise niedrigen Entlohnung einhergeht, ist zudem für die relativ beschäftigungsstarken (zu erkennen an der Größe des Kreises) Bereiche der Fahrzeugführung im Straßenverkehr sowie der Körperpflege festzustellen. Für die Mehrzahl der Berufsgruppen liegt der Corona-Effekt hingegen vergleichsweise nah am Median (dargestellt durch die horizontale gestrichelte Linie).

Wir untersuchen zudem den Zusammenhang zwischen der Höhe des Corona-Effekts und dem berufsspezifischen Medianentgelt durch die Schätzung eines linearen Regressionsmodells. Über alle Berufsgruppen hinweg zeigt sich, dass eine geringere Entlohnung im Durchschnitt mit einem höheren krisenbedingten Anstieg der Zugänge in Arbeitslosigkeit einhergeht. Der Zusammenhang ist jedoch nur schwach signifikant. Vergleicht man Berufe, deren Medianentgelt um 1.000 Euro auseinanderliegt, dann werden für die geringer entlohnten Berufsgruppen durchschnittlich 1,8 zusätzliche Übergänge aus Beschäftigung in Arbeitslosigkeit pro 1.000 sozialversicherungspflichtig Beschäftigte erwartet.

Abbildung 2 verdeutlicht jedoch, dass der Zusammenhang zwischen Betroffenheit und Entgelt nicht einheitlich ist. Während geringer entlohnte Berufsgruppen in der unteren Hälfte der Medianentgeltverteilung (links der vertikalen gestrichelten Line) stärker betroffen sind, findet sich in der oberen Hälfte (rechts der Linie) kein entsprechender Zusammenhang. Diesen Unterschied verdeutlichen die beiden Regressionsgeraden: Der Wert von -0,0075 für den Zusammenhang zwischen Corona-Effekt und Medianentgelt im unteren Bereich der Lohnverteilung bedeutet, dass für Berufsgruppen in diesem Segment, deren Medianentgelt um 1.000 Euro geringer ausfällt, 7,5 zusätzliche Übergänge in Arbeitslosigkeit erwartet werden. Im Gegensatz dazu ist ein negativer Zusammenhang im oberen Teil der beruflichen Lohnverteilung nicht zu erkennen. Mit anderen Worten: Innerhalb der Gruppe der besser entlohnten Arbeitskräfte geht ein niedrigeres Entgelt nicht mit einem höheren Anstieg der Übergänge in Arbeitslosigkeit einher. Insgesamt zeigt sich, dass die Berufsgruppen nicht einheitlich von höheren Übergängen in Arbeitslosigkeit betroffen sind, sondern dass insbesondere Berufe am unteren Ende der Lohnverteilung eine höhere Betroffenheit aufweisen.

## Wie verändert sich der Zusammenhang zwischen Corona-Effekt und Entlohnung im Zeitverlauf?

Die Auswirkungen der Corona-Pandemie auf das Wirtschaftsleben haben sich im Zeitverlauf verändert. Nach einem umfassenden Lockdown im April konnten wirtschaftliche Aktivitäten im Zuge sinkender Neuinfektionszahlen im Frühsommer häufig wiederaufgenommen bzw. ausgeweitet werden. Angesichts des neuerlichen Anstiegs der Infektionen im Herbst wurde seit Anfang November das Wirtschaftsleben zunächst in bestimmten Bereichen wie der Unterhaltung oder der Gastronomie erneut stark eingeschränkt. Wie Böhme et al. (2020) zeigen, variieren mit diesen Maßnahmen auch die coronabedingten Arbeitsmarkteffekte im Zeitverlauf: Nach starken Kriseneffekten im April und Mai waren in den Folgemonaten gewisse Erholungstendenzen festzustellen.

Angesichts dieses zeitlichen Musters stellt sich die Frage, ob der Zusammenhang zwischen Corona-Effekt und Entlohnung ebenfalls im Zeitverlauf variiert. Waren also geringer entlohnte Berufe in der unteren Hälfte der Lohnverteilung durchgehend stärker von Übergängen in Arbeitslosigkeit betroffen, oder ist dies nur für einzelne Monate zu beobachten? Um diese Frage zu beantworten, replizieren wir die in Abbildung 2 dargestellte Untersuchung separat für die einzelnen Monate im Zeitraum April bis November 2020. Abbildung 3 zeigt für jeden Monat den geschätzten Zusammenhang zwischen dem Corona-Effekt und der Entlohnung für die untere (dunkelblau) und die obere Hälfte der Lohnverteilung (hellblau). Mit dieser Analyse wird auch die Bedeutung der einzelnen Monate für den Zusammenhang im Gesamtzeitraum erkennbar: Die Ergebnisse für die einzelnen Monate summieren sich für den unteren Bereich der Entgeltverteilung zum Wert von -0,0075 auf, dem für den

Abbildung 3

**Zusammenhang zwischen dem Corona-Effekt und dem Medianentgelt nach Monaten**

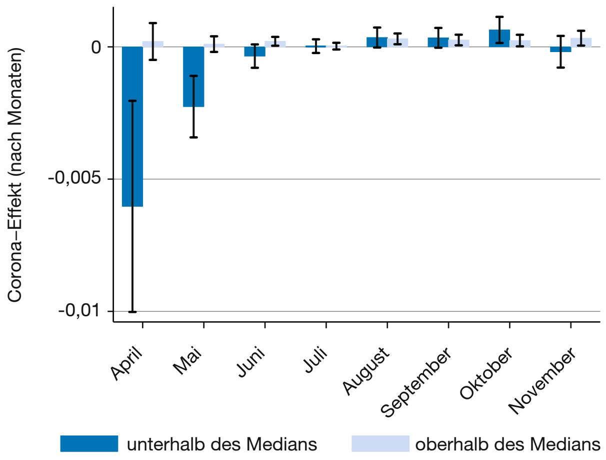

Anmerkungen: Dargestellt ist der geschätzte Zusammenhang zwischen dem monatlichen Corona-Effekt und dem Medianentgelt für Berufsgruppen unterhalb bzw. oberhalb des mittleren Medianentgelts. Es werden robuste Standardfehler berechnet. Die durchgezogenen Linien stellen das 95 %-Konfidenzintervall dar.

Quelle: Statistik der Bundesagentur für Arbeit; eigene Berechnungen.

Gesamtzeitraum ermittelten Zusammenhang (dies gilt analog für den oberen Bereich der Lohnverteilung).

Abbildung 3 zeigt, dass die stärkere Krisenbetroffenheit gering entlohnter Berufsgruppen in der unteren Hälfte der Lohnverteilung vor allem auf die Monate April und Mai zurückzuführen ist, in denen es im Zuge der Einschränkungen des Wirtschaftslebens zu einem starken Anstieg der Übergänge in Arbeitslosigkeit gekommen ist. Im Juni und Juli findet sich dagegen kein signifikanter Zusammenhang mehr zwischen dem Corona-Effekt und der Höhe des Entgelts. Zwischen August und Oktober dreht sich das Vorzeichen des Zusammenhangs um. Das bedeutet, dass sich die Zugänge in Arbeitslosigkeit gegenüber dem Vorjahr für geringer entlohnte Berufsgruppen günstiger entwickelt haben als für höher entlohnte. Diese Entwicklung setzt sich jedoch im November nicht fort. Ob mit den neuerlichen Einschränkungen des Wirtschaftslebens abermals jene Berufsgruppen stärker von Übergängen in Arbeitslosigkeit betroffen sein werden, die auch zu Beginn der Krise eine höhere Betroffenheit aufwiesen, bleibt abzuwarten.

Im oberen Teil der Lohnverteilung fällt der Zusammenhang zwischen Corona-Effekt und Entgelt vergleichsweise schwach aus und ändert sich im Zeitverlauf nur wenig. Darin spiegelt sich auch wider, dass die besser entlohnten Arbeitskräfte in Deutschland im Mittel in deutlich geringerem Maße den krisenbedingten Ausschlägen am Arbeitsmarkt ausgesetzt waren als die Beschäftigten im unteren Lohnbereich.

## Ausblick

Die Ergebnisse unserer Analyse deuten darauf hin, dass sich die soziale Ungleichheit in Deutschland durch die Arbeitsmarkteffekte der Corona-Krise verschärfen könnte. So sind Berufe aus dem unteren Einkommenssegment, wie beispielsweise Berufe in der Hotellerie oder im Gastgewerbe, deutlich stärker durch den coronabedingten Arbeitsmarktschock betroffen als besser entlohnte Berufsgruppen. Auch andere Untersuchungen zeigen, dass von den wirtschaftlichen Folgen der Pandemie häufig jene Arbeitskräfte überdurchschnittlich betroffen sind, die schon vor der Krise relativ ungünstig am Arbeitsmarkt positioniert waren – nicht nur in Bezug auf ihre Entlohnung, sondern beispielsweise auch im Hinblick auf das Arbeitslosigkeitsrisiko oder die Stabilität von Beschäftigungsverhältnissen. So erleiden vor allem Erwerbspersonen mit Migrationshintergrund, geringfügig oder befristet Beschäftigte sowie Leiharbeitskräfte vergleichsweise häufig Einkommensverluste durch die Corona-Krise (Hövermann und Kohlrausch 2020). Zudem ist festzustellen, dass Beschäftigte in finanziell besser gestellten Haushalten seltener von Kurzarbeit betroffen sind (Kruppe und Osiander, 2020).

Da durch den erneuten Lockdown im Winter überwiegend die gleichen Wirtschaftsbereiche wie im Frühjahr betroffen sind, ist zu erwarten, dass die damit verbundenen Arbeitsmarkteffekte wiederum vor allem Arbeitskräfte mit geringen Löhnen treffen. Sollte sich abzeichnen, dass sich infolge der Corona-Krise soziale Ungleichheiten in Deutschland dauerhaft zu verschärfen drohen, ergibt sich auch daraus ein Handlungsauftrag an die Politik. Eine Reihe von steuer- und transferpolitischen Maßnahmen sowie die umfassenden Kurzarbeiterregelungen wirken bereits einkommensstabilisierend, insbesondere auch für einkommensschwache Haushalte (Bruckmeier et al., 2020). Darüber hinaus sind verstärkt jene Arbeitskräfte in den Blick zu nehmen, die durch den Lockdown bereits im Frühjahr ihren Arbeitsplatz verloren haben und von der Arbeitsmarkterholung im Sommer nicht profitieren konnten. Mit zunehmender Dauer der Arbeitslosigkeit und der Krise sinken die Aussichten auf eine schnelle Reintegration dieser Erwerbspersonen in den Arbeitsmarkt. Damit gewinnen insbesondere bedarfsgerechte Fort- und Weiterbildungsmaßnahmen an Bedeutung, um die Chancen für eine dauerhafte Beschäftigung zu verbessern und Wechsel in höher entlohnte Berufe zu erleichtern. Weiterhin ist zu erwarten, dass nach der Krise der Fachkräftebedarf mit seinen wachsenden und sich wandelnden Qualifikationsanforderungen wieder stark zunimmt. Dies gilt umso mehr vor dem Hintergrund, dass die gegenwärtige Krise möglicherweise als Katalysator für längerfristig wirkende Prozesse des Strukturwandels am Arbeitsmarkt wirkt (Nitt-Drießelmann et al., 2020). Auch von strukturellen Veränderungen wie der Digitalisierung sind häufig gering qualifizierte Arbeitskräfte mit geringem Einkommen überdurchschnittlich betroffen.



Literatur

Böhme, S., C. Burkert, J. Carstensen, L. Eigenhüller, A. Niebuhr, D. Roth, G. Sieglen und D. Wiethölter (2020), Die Bedeutung der regionalen Wirtschaftsstruktur für die Arbeitsmarkteffekte der Corona-Pandemie — Eine erste Einschätzung, IAB-Forschungsbericht, 15/2020.
Bruckmeier, K., A. Peichl, M. Popp, J. Wiemers und T. Wollmershäuser (2020), Distributional Effects of Macroeconomic Shocks in Real-Time: A Novel Method Applied to the Covid-19 Crisis in Germany, IAB-Discussion Paper, 36/2020.10.1007/s10888-021-09489-4 PMC8452132 34566543
Gehrke, B. und E. Weber (2020), Kurzarbeit, Entlassungen, Neueinstellungen: Wie sich die Corona-Krise von der Finanzkrise 2009 unterscheidet, IAB-Forum, 28. Mai 2020.
Hövermann A Kohlrausch B Soziale Ungleichheit und Einkommenseinbußen in der Corona-Krise — Befunde einer Erwerbstätigenbefragung WSI Mitteilungen 2020 6 485 492 10.5771/0342-300X-2020-6-485
Kruppe, T. und C. Osiander (2020), Kurzarbeit in der Corona-Krise: Wer ist wie stark betroffen?, IAB-Forum, 30. Juni 2020.
Nitt-Drießelmann, D., A. Lagemann, K. Nau und A. Wolf (2020), Arbeitslosigkeit bei Gering- und Mittelqualifizierten im Zuge der COVID-19-Pandemie: Eine Analyse für ausgewählte Berufsgruppen, HWWI Policy Paper, 129.
